# A semi‐automated pipeline for quantitation of Pax7+, myonuclei, and cross‐sectional area by fiber type

**DOI:** 10.14814/phy2.71097

**Published:** 2026-09-21

**Authors:** Helia G. Megowan, Madeline Luu, Adam Shuaib, Kaitlyn B. Augienello, Adam C. Fries, Jake Searcy, Hans C. Dreyer

**Affiliations:** ^1^ Department of Human Physiology University of Oregon Eugene Oregon USA; ^2^ Department of Computer Science University of Oregon Eugene Oregon USA; ^3^ Department of Data Science University of Oregon Eugene Oregon USA; ^4^ Department of Neuroscience University of Oregon Eugene Oregon USA; ^5^ Genomics & Cell Characterization Core Facility University of Oregon Eugene Oregon USA

**Keywords:** automated analysis, CellProfiler, muscle stem cells, skeletal muscle

## Abstract

Manual analysis of skeletal muscle cross‐sections is time‐consuming and subject to error and user bias. To overcome these limitations, we developed and validated a semi‐automated, quantitative, and reproducible image‐analysis pipeline specifically tailored to quantify Pax7+ satellite cells, myonuclei, and cross‐sectional area by fiber type. The workflow combines Fiji/ImageJ‐based image preprocessing with CellProfiler, Cellpose, and a custom Python script to process and analyze immunohistological images of muscle tissue cross‐sections. Outcomes include Pax7+ satellite cells and myonuclei quantified per fiber by fiber type, along with cross‐sectional area, perimeter, and fiber type classification. This semi‐automated approach provides a robust and efficient platform for high‐throughput analysis of muscle tissue cross‐sections from large datasets.

## Graphical abstract

Created in BioRender. Megowan, H. (2026) https://BioRender.com/9457v5e.

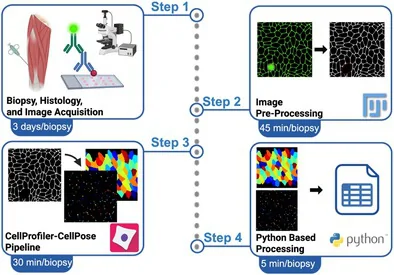

## 1 INTRODUCTION

Histological evaluation of skeletal muscle reveals how tissue structure relates to baseline physiology and experimental responses (Mazzarini et al., 2021). Immunohistochemistry, using labeled antibodies to detect specific proteins, combined with high‐resolution imaging, maps the cellular and subcellular organization of muscle. Muscle fibers are highly plastic—showing altered cross‐sectional area (CSA), shifts in myonuclear number and position, and modulation of Pax7+ satellite cell pools—in response to internal and external cues. These histological endpoints are routinely used as structural surrogates for muscle functional capacity and adaptive potential.

Precise quantification of Pax7+ satellite cells is essential for understanding of skeletal muscle adaptations, including myonuclear accretion. Satellite cells give rise to new myonuclei, making them relevant to the ongoing assessment of the myonuclear domain hypothesis, which posits that each myonucleus regulates a defined cytoplasmic volume (Cheek et al., 1971; Cramer et al., 2020; Hansson et al., 2020). Emerging data indicate the domain may align more closely with fiber perimeter than with cross‐sectional area, supporting use of perimeter as an outcome (Moesgaard et al., 2022). Further, accurately quantifying myonuclei per muscle cell is important for monitoring the response or lack thereof to various stimuli. Taken together, these measures provide mechanistic insight into muscles' responses to interventions, providing foundational evidence for their use in clinical applications (Muyskens et al., 2019).

A common approach quantifies muscle structural changes on cross‐sections cut perpendicular to fiber long axes, which samples many individual fibers (each fiber is a single muscle cell) and therefore better represents the whole muscle. Traditional manual counting (commonly targeting ~50 type I and ~75 type II fibers for adequate power (Mackey et al., 2009)) is feasible for small datasets but is laborious and error‐prone when biopsies yield hundreds to >1000 fibers. Automated image analysis enables scalable, reproducible quantification of large cross‐sections, yet existing tools (Babcock et al., 2020; Encarnacion‐Rivera et al., 2020; Lau et al., 2018; Mayeuf‐Louchart et al., 2018; Sanz et al., 2019; Wen et al., 2018) often measure variables relevant to a specific study (such as capillaries) and can be difficult to adapt across antibodies, imaging settings, or file formats.

Motivated by the above, in this paper, we developed a semi‐automated image analysis pipeline for immunohistological images of skeletal muscle cross‐sections that quantifies:

1. Myonuclear number per muscle cell
2. Pax7+ number per muscle cell
3. Fiber type of each muscle cell
4. Cross‐sectional area of each muscle cell
5. Perimeter of each muscle cell

This study is, to our knowledge, the first study to combine these five analytic targets into a single analysis pipeline compatible with standard widefield microscopy—a widely available platform in core facilities and individual laboratories. This workflow enables accurate, high‐throughput quantification of muscle cross‐sections while preserving full visual outputs for user review and quality control. The method has broad applicability in biomedical research and may help uncover mechanisms related to satellite cells and myonuclei within various physiological and pathological contexts across the lifespan.

## 2 METHODS

### 2.1 Ethical approval

The study was approved by the University of Oregon Institutional Review Board and conducted in accordance with *Declaration of Helsinki* ethical principles. All subjects gave informed written consent prior to study participation.

### 2.2 Biopsies

Skeletal muscle biopsies were performed on human vastus lateralis in a distal to proximal sequence and in the fasted state. For serial biopsies, the biopsy needle was oriented lateral from and perpendicular to the long axis of the femur, with the biopsy needle window/opening situated in the mid‐substance of the muscle tissue. Serial biopsies were thus obtained from the mid‐substance of the vastus lateralis muscle that are 3 or more cm apart (and not angled) (Højfeldt et al., 2025; Mackey et al., 2016). Biopsy samples were obtained using a Bergström needle with a 5 mm internal diameter with manual suction. Each biopsy yielded approximately 300 mg of tissue (range ~100–500 mg) with the fiber bundles approximating 2–7 mm in length.

### 2.3 Immunohistochemistry

Immediately following biopsy, tissue was trimmed of fat and connective tissue, aligned longitudinally, embedded in Optimal Cutting Temperature compound (OCT), and flash frozen in liquid nitrogen‐cooled isopentane. To flash freeze, a metal beaker with isopentane was submerged in liquid nitrogen until frozen (freezing point of isopentane: −160°C). The center of the frozen isopentane was thawed and tissue was submerged into the liquid‐phase isopentane (2‐methylbutane), flash frozen, and stored in a cryovial at −80°C.

**FIGURE 1:**
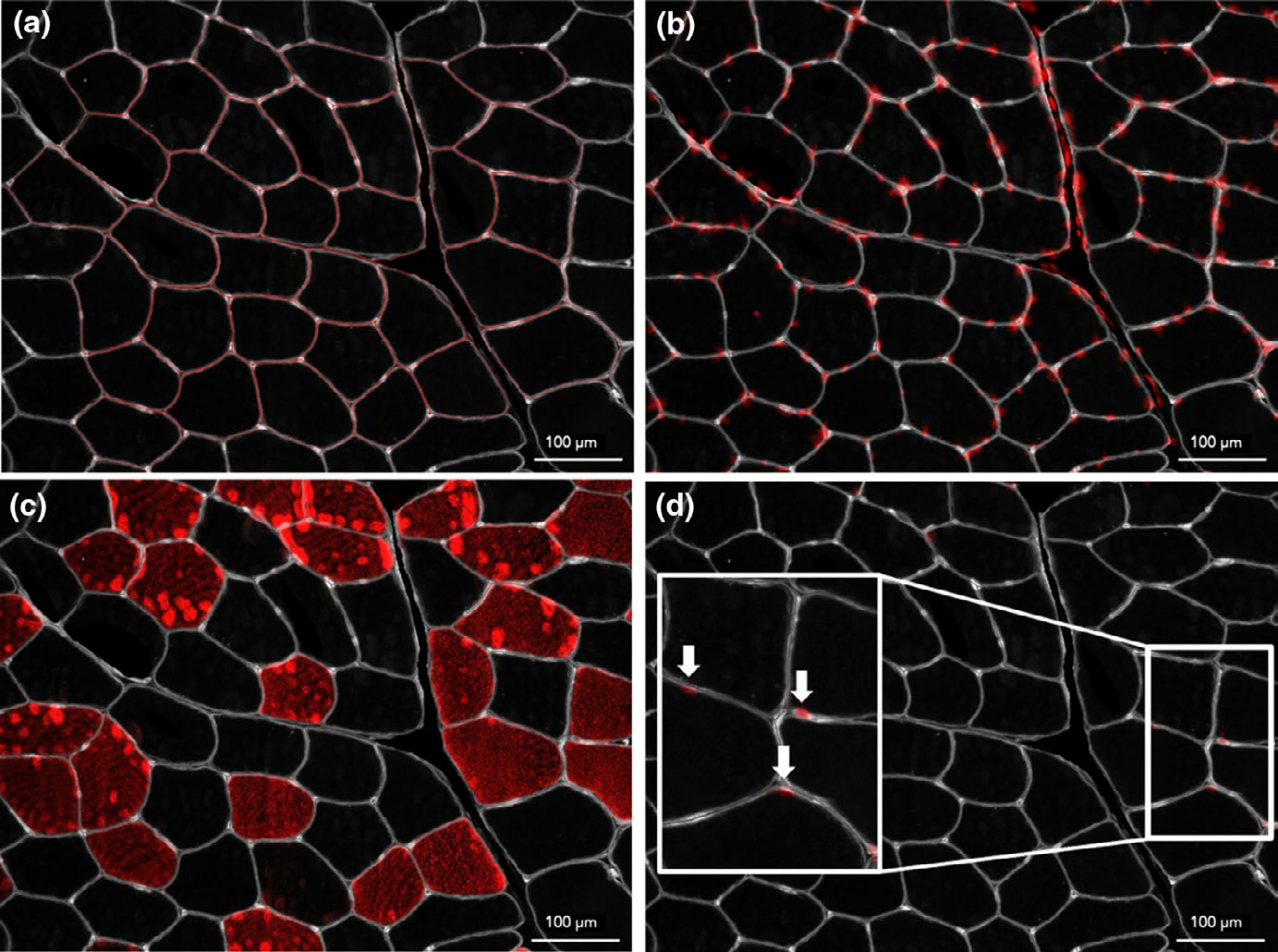
*Vastus lateralis* cross‐section histology. Laminin stain is shown in white, and parameters of interest are shown in red. Images taken at 20×. Scale bars = 100 μm. (a) Red overlay shows segmentation of myofibers by CellProfiler, after image pre‐processing. (b) DAPI. (c) Myosin Heavy Chain I indicating type I fibers. (d) White arrows within callout indicate Pax7+ satellite cells.

Tissue sections were cut at 7 μm on a Leica CM 1850UV cryostat set to −25°C and air dried for 1 h at room temperature protected from light. ImmEdge Hydrophobic Barrier (PAP) pen (cat. no. H‐4000, Vector Laboratories, Newark, CA, USA) was used to draw a border around the tissue sections and allowed to dry for at least 5 min. Sections were fixed in ice cold acetone (stored at −20°C) for 3 min on the bench and washed in PBS (3 × 5 min washes). Sections were then blocked with 3% H_2_O_2_ for 7 min (placed on the slide), washed in PBS (3 × 5 min washes), and blocked for 1 h at room temperature in blocking buffer. Blocking buffer consisted of 5% normal goat serum (cat. no. 005‐000‐121, Jackson ImmunoResearch, West Grove, PA, USA), 5% normal donkey serum (cat. no. 017‐000‐121, Jackson ImmunoResearch, West Grove, PA, USA), and 1% glycine in PBS. Primary antibodies for laminin (1:200 Anti‐Laminin, cat. no. L9393, Sigma‐Aldrich, Darmstadt, Germany, Figure 1a), type I myosin heavy chain (1:75 BA‐D5‐c, deposited to the DSHB by Schiaffino, S., DSHB Hybridoma Product BA‐D5, Figure 1c), and satellite cells (1:100 PAX7‐c, deposited to the DSHB by Kawakami, A., DSHB Hybridoma Product PAX7, Figure 1d) were mixed in blocking buffer, allowed to incubate for 1 h at room temperature, and then moved to 4°C for overnight incubation. Primary antibodies and concentrations can be found in Table 1.

**TABLE 1:** Primary and secondary antibodies.

Target	Primary antibody	Concentration	Manufacturer	Secondary antibody	Concentration	Manufacturer
Laminin	Anti‐Laminin	1:200	Sigma Aldrich, L9393	Alexa Fluor 647 IgG	1:500	Invitrogen, A21245
Type 1 Myosin Heavy Chain	BA‐D5‐c	1:75	DSHB, BA‐D5	Cy3 IgG	1:250	Jackson ImmunoResearch,715‐165‐150
Pax7+ Satellite Cells	PAX7‐c	1:100	DSHB, PAX7	Biotin‐SP IgG; Alexa Fluor 488	1:1000; 1:200	Jackson ImmunoResearch,115‐065‐205; Invitrogen, B40932

After overnight incubation, sections were washed in PBS (3 × 30 min washes), blocked with 3% H_2_O_2_ for 7 min, and washed again in PBS (3 × 3 min washes). Secondary antibodies for laminin (1:500 Alexa Fluor 647 IgG, cat. no. A21245, Invitrogen, Carlsbad, CA, USA), type I myosin heavy chain (1:250 Cy3 IgG, cat. no. 715‐165‐150, Jackon ImmunoResearch, West Grove, PA, USA), and satellite cells (1:1000 Biotin‐SP IgG, cat. no. 115‐065‐205, Jackon ImmunoResearch, West Grove, PA, USA) were mixed in blocking buffer and incubated for 1 h at room temperature. Secondary antibodies and concentrations can be found in Table 1. Following secondary incubation, sections were washed in PBS (3 × 3 min washes) and incubated for 1 h at room temperature in HRP‐conjugated streptavidin (cat. no. B40932, Invitrogen, Carlsbad, CA, USA). Sections were then washed in PBS (3 × 3 min washes) and incubated for 1 h at room temperature in Alexa Fluor 488 (1:200 in PBS, cat. no. B40932, Invitrogen, Carlsbad, CA, USA). Finally, sections were washed in PBS (3 × 3 min washes) and mounted with SlowFade Diamond Antifade with DAPI (cat. no. S36964, Invitrogen, Carlsbad, CA, USA, Figure 1b).

### 2.4 Image acquisition

Tissue sections were imaged on a Leica DM4000B widefield fluorescence microscope with a Leica DFC360FX monochrome camera (pixel size 6.45 × 6.45 μm) and a Lumen200 metal halide lamp, (Prior Scientific, Cambridge, United Kingdom). Images were acquired at 20× magnification (HC PL FLUOTAR 20×/0.50; 1pixel = 1.9535 μm) in four channels: DAPI for nuclei (blue) (Ex: 340‐380 nm, Em: 425 nm), FITC/GFP for Pax7+ satellite cells (green) (Ex: 492 nm, Em: 520 nm), TRITC/Cy3 for type I myosin heavy chain (yellow) (Ex: 550 nm, Em: 570 nm), and Cy5 for laminin (far red) (Ex: 650 nm, Em: 670 nm). Exposure times, camera gain, and light intensity were adjusted for each channel to maximize dynamic range without saturation. In our hands, exposure times typically range between 100 and 300 ms; however, these settings should be empirically determined by each user depending on imaging system (Murach et al., 2019). Multiple non‐overlapping fields of view were collected systematically across each section.

### 2.5 Image cleaning and pre‐processing

**FIGURE 2:**
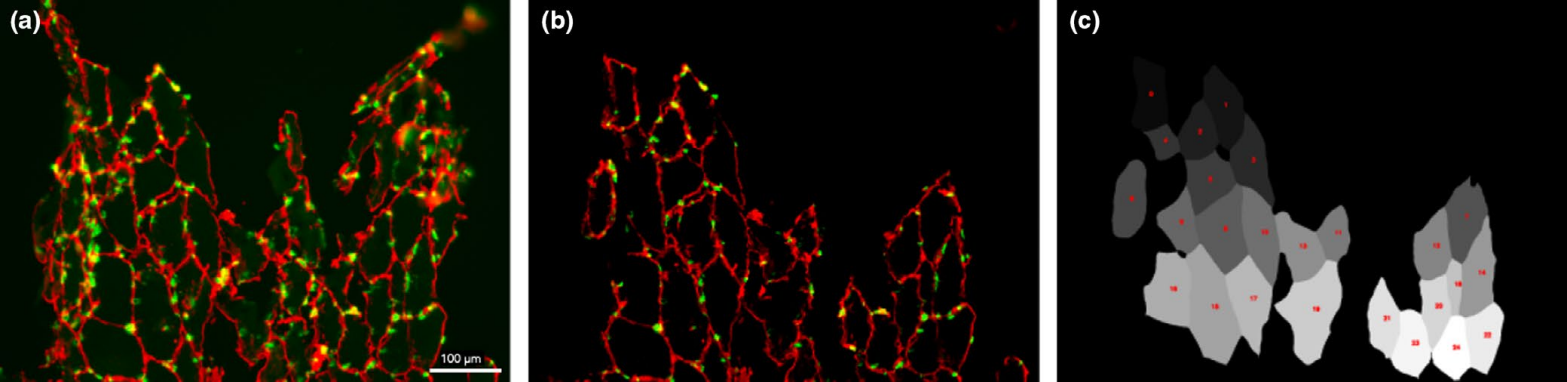
Imperfect images can be segmented by the program with pre‐processing. Red staining shows laminin (cell borders) and green staining shows DAPI (nuclei). Scale bar = 100 μm. (a) Raw image overlay of the imperfect edge of a tissue section at 20×. (b) The same image overlay post‐cleaning in Fiji. (c) Labeled cells identified by the pipeline from tissue section shown in a.

To ensure the integrity of the automated analysis pipeline and maximize the reliability of downstream data, a standardized image “cleaning” protocol was implemented (Figure 2). The primary objective of this pre‐processing stage was the systematic removal of optical artifacts and abnormal regions—such as overlapping or blurred laminin signals and out of focus or extraneous nuclei—that could potentially introduce noise or lead to false‐positive identifications within the CellProfiler environment. The “cleaned” images better reflect the high‐fidelity regions measured in manual, by‐hand analysis.

The workflow utilizes Fiji/ImageJ for manual curation and signal enhancement (Schindelin et al., 2012). Signal‐to‐noise ratio is first optimized through individual channel brightness and contrast adjustments; specifically, the “Minimum” and “Maximum” toggles were visually calibrated to enhance feature visibility.

After brightness and contrast adjustments have been applied to individual images, the laminin, DAPI, and Pax7 channels are merged into a composite image using the “Merge Channels” function, ensuring “Keep source images” was enabled to preserve the underlying raw data. Areas identified as problematic—including non‐specific staining, tissue folds, blood vessels, or poor nuclear definition—were manually circumscribed and removed using the “Clear” function.

Following artifact removal, the composite images were decomposed into their constituent channels (“Split Channels”) and exported as 8‐bit .tif files into one folder containing all images for that sample. To ensure compatibility with the automated modular architecture of the CellProfiler‐Cellpose integration, a strict naming convention was applied (e.g., Subject8_BL_L_Set1_Lam), facilitating the seamless batch processing and metadata assignment required for the final morphometric analysis.

### 2.6 CellProfiler‐Cellpose pipeline

We developed a custom CellProfiler‐Cellpose pipeline to process cleaned immunohistochemistry images. This workflow automates 2D cellular and myonuclear segmentation, feature extraction, and file organization, yielding binarized mask outputs as well as visual overlays for muscle fibers, myonuclei, and satellite cells.

CellProfiler (v4.2.8) is an open‐source software tool for automated image analysis, currently developed and maintained in Cimini Lab at the Broad Institute of MIT and Harvard (Stirling et al., 2021). CellProfiler is designed for high‐throughput, quantitative microscopy workflows, using modular pipelines for object segmentation and measurement.

The pipeline integrates Cellpose (v2.3.2), a machine learning model plugin developed by Carsen Stringer and Marius Pachitariu at the Howard Hughes Medical Institute (HHMI) Janelia Research Campus (Stringer et al., 2021). Cellpose uses a U‐Net–style convolutional neural network to predict likely cell regions and to reconstruct cell boundaries. Cellpose was selected for myonuclear segmentation as its pretrained models accurately segment diverse tissue morphologies without the need for custom training. This compatibility facilitates the automated segmentation of irregular and overlapping objects within a high‐throughput framework. Further information on the Cellpose algorithm can be found here: https://doi.org/10.1038/s41592‐020‐01018‐x.

The pipeline operates across two computing environments: a standard Windows desktop workstation and a high‐performance supercomputer. Deployment occurs either through Docker (v28.2.2) or within a local Python environment. Docker provides enhanced stability on desktop systems, while native Python execution yields superior performance on high‐performance clusters. For further computing specifications, see supplemental.

Careful reading of attached supplemental documents and the CellProfiler manual (https://cellprofiler‐manual.s3.amazonaws.com/CellProfiler‐4.2.8/index.html) is encouraged before using the pipeline. As highlighted in the manual, for each module within the CellProfiler pipeline, there are several adjustable variables that will impact the way the module functions. Below, we have listed the values that were found to be most accurate for our images within this pipeline, but users should test and adjust these variables to tailor it to their images specifically.

#### 2.6.1 Overview of the CellProfiler pipeline

##### Metadata extraction and image organization

The *Metadata* module utilizes Regular Expression patterns to dynamically group and extract metadata, including Subject ID, biopsy timepoint, leg (L/R), image set, and staining protocol. The *NamesAndTypes* module identifies specific stains using the following tags:

- *Laminin*: laminin
- *DAPI*: nuclei
- *Pax7*: satellite
- *MyHC1*: myhc1

The *Groups* module then organizes input image sets by *“Subject”, “Day”, “Side”*, and *“Set”*, as extracted by the *Metadata* module.

##### Cellular and myonuclear segmentation

**FIGURE 3:**
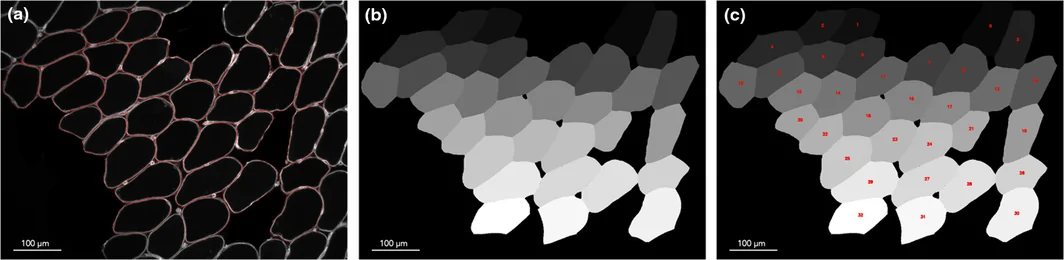
Myofiber segmentation with Cellpose “cyto2” detection model. (a) White staining shows fluorescent laminin signal, with red overlay showing cells accepted by the pipeline. This overlay is saved for each image set to allow for manual quality control. Scale bar = 100 μm. (b) Grayscale object masks generated and saved by the pipeline for use in the Python script. Scale bar = 100 μm. (c) Masks identified by the pipeline are numbered by the Python script so that each cell within each image has a unique identifier, allowing for manual quality controls. Scale bar = 100 μm.

**FIGURE 4:**
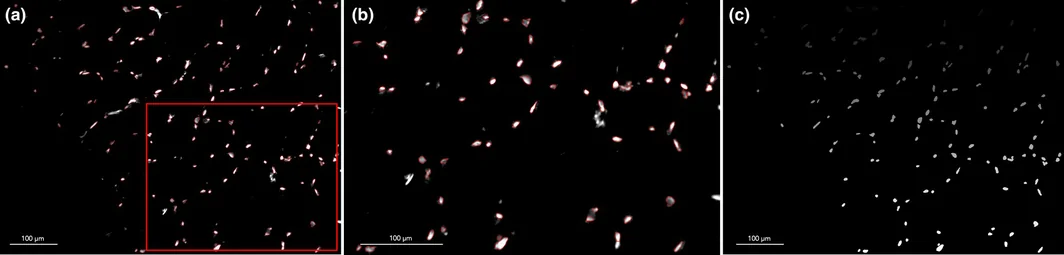
Nuclear segmentation with Cellpose “nuclei” detection model, after second iteration of *RunCellpose* module. (a) White staining shows fluorescent DAPI signal, with red overlay showing nuclei accepted by the pipeline. This overlay is saved for each image set to allow for manual quality control. Scale bar = 100 μm. (b) Callout from red box in (a). Scale bar = 100 μm. (c) Grayscale object masks generated and saved by the pipeline for colocalization with myofibers in the Python script. Scale bar = 100 μm.

For cellular segmentation, the pipeline employs the “cyto2” detection mode within the Cellpose module on laminin‐stained images to identify sarcolemmal boundaries (Figure 3). For myonuclear segmentation (Figure 4), the workflow initiates with a *GaussianFilter* (sigma = 3) to smooth the DAPI signal and reduce noise. To address the high sensitivity of the default *Cellpose* “nuclei” model, the pipeline implements an iterative subtraction logic to minimize false‐positive detections:

1. *Initial detection*: A *RunCellpose* module invokes the “nuclei” model to identify primary nuclear candidates (flow_threshold = 0.4; cellprob_threshold = 0.0).
2. *Background normalization*: The *MeasureObjectIntensity* module calculates the current object intensity distribution, and the *ImageMath* module subtracts the 17th percentile of pixel intensities from the original DAPI signal. This suppresses low‐level background fluorescence and non‐specific staining.
3. *Refined segmentation*: The cleaned image passes through a second *RunCellpose* module with optimized parameters to ensure precise boundary adherence.

##### Satellite cell segmentation

**FIGURE 5:**
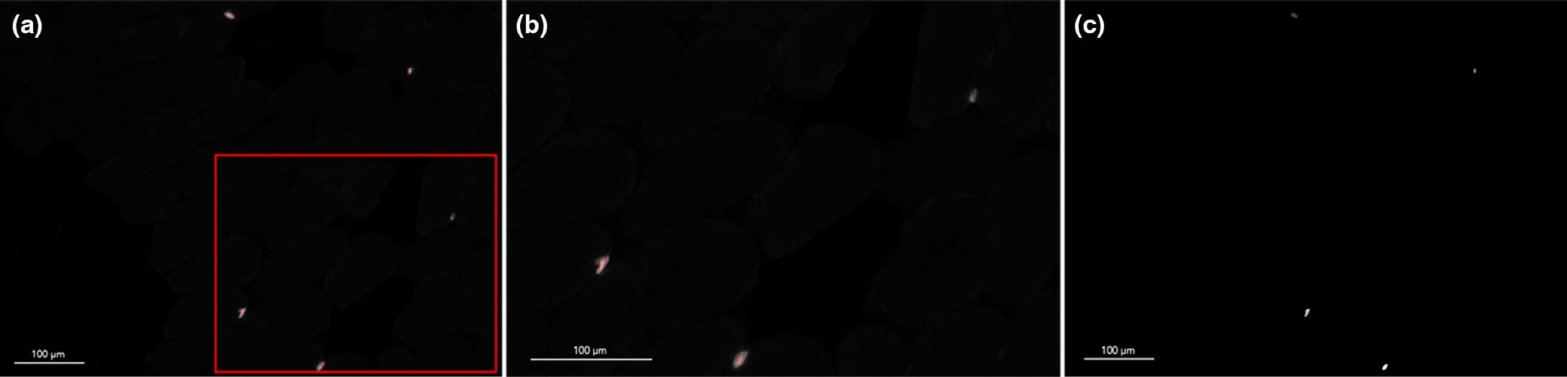
Satellite cell segmentation with *IdentifyPrimaryObjects* module. (a) White staining shows fluorescent Pax7+ signal, with red overlay showing satellite cells accepted by the pipeline. This overlay is saved for each image set to allow for manual quality control. Scale bar = 100 μm. (b) Callout from red box in (a). Scale bar = 100 μm. (c) Grayscale object masks generated and saved by the pipeline for colocalization with myofibers in the Python script. Scale bar = 100 μm.

Pax7+ satellite cell segmentation utilizes the *IdentifyPrimaryObjects* module (Figure 5), as deep‐learning architectures prove insufficient for resolving sparse Pax7 signals against the sarcoplasmic background. The module employs an Otsu thresholding strategy to isolate satellite cells within a defined pixel diameter range of 16–60; this parameter can be adjusted based on the expected size of objects of interest. Objects are further filtered by circularity and intensity to exclude signal artifacts.

The pipeline is configured for high‐throughput batch processing. By leveraging the *Metadata* and *Groups* modules, the system systematically organizes segmentation masks and morphological data into a standardized hierarchical directory structure. This consistent naming convention allows a downstream Python‐based analysis script to programmatically parse and aggregate data for final statistical refinement.

### 2.7 Python‐based processing

To transition from raw segmentation masks to refined, biologically relevant datasets, we developed a custom Python‐based processing pipeline utilizing the OpenCV, pandas, and NumPy libraries. The pipeline is designed to automate the integration of multi‐channel spatial data, specifically matching nuclear and satellite cell positions to individual muscle fiber domains. This stage of the workflow (1) ensures objective fiber‐type assignment, (2) quantifies morphological parameters in physical units, and (3) aggregates data at the biopsy level while maintaining full traceability of individual fibers.

#### 2.7.1 Input structure and data parsing

The pipeline employs a dynamic directory parser that identifies image sets based on a standardized naming convention. The script scans designated subdirectories for four critical components:

- *Laminin masks*: Grayscale images where each fiber is represented by a unique pixel intensity value
- *Nuclei masks*: Binary/grayscale masks representing DAPI‐stained nuclei.
- *Satellite cell masks*: Binary/grayscale masks representing Pax7‐stained cells.
- *MyHC1 images*: Raw fluorescence images used for fiber‐type intensity quantification.

#### 2.7.2 Grayscale mask decomposition

Because Cellpose segmentation outputs a single label matrix where each object has a unique grayscale value, the function *split_mask_by_gray_level* was implemented. This function iterates through the unique pixel values of the image, extracting each identified fiber or nucleus into an individual binary mask. This decomposition is essential for the subsequent bitwise overlap analysis and morphological measurements.

#### 2.7.3 Fiber‐object assignment

To accurately count nuclei and satellite cells per fiber, we developed a “greatest overlap” assignment logic via the *overlap_with_objects* and *fiber_assign* functions.

*Bitwise intersection*: For every fiber mask, the pipeline performs a bitwise AND operation against all identified object masks (nuclei or satellite cells).

*Primary assignment*: To account for objects that may partially overlap with multiple fibers, each object is strictly assigned to the fiber with which it shares the largest number of overlapping pixels.

*Quantification*: This results in a discrete count of myonuclei and satellite cells localized specifically to each fiber's cytoplasmic domain.

#### 2.7.4 Morphometry and scaling

Fiber area and perimeter are calculated using the *calculate_area_and_perimeter* function. To ensure data is reported in biological units, a pixel‐to‐micron scaling factor is applied (1.9535 pixels/micron for 20× magnification).

#### 2.7.5 Fiber type assignment

**FIGURE 6:**
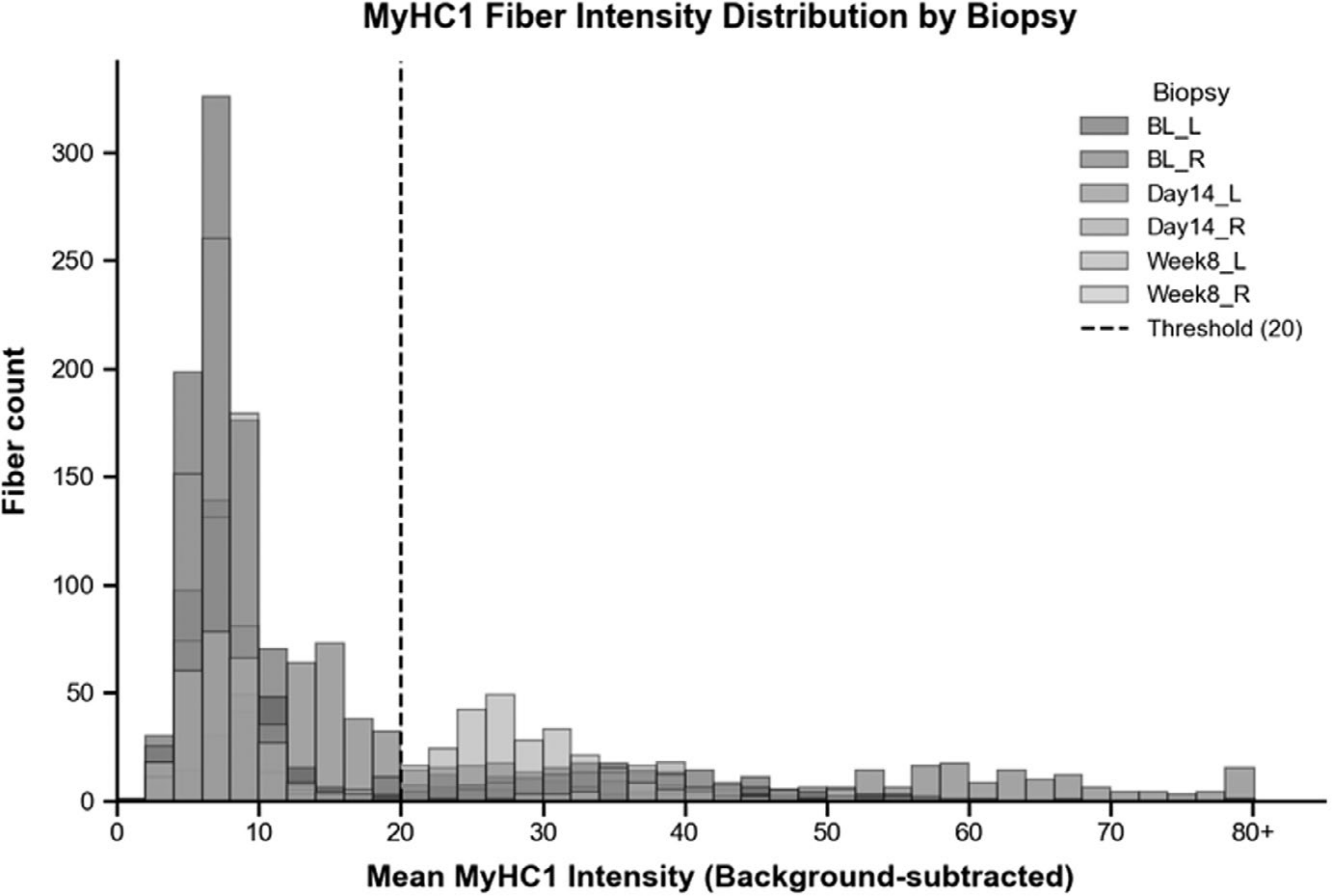
Intensity threshold for automated fiber typing. Analysis of intensities within segmented myofibers from 3722 muscle cells from 12 human biopsies was used to establish a threshold between type I and type II fibers. The frequency distribution shows two groups, with type II fibers being the group below an intensity of 20 and type I fibers being the group above an intensity of 20. The dotted line represents the threshold at pixel value 20.

The pipeline automates the classification of type I and type II fibers using MyHC1 fluorescence. Background is removed from images with the function *background_subtract* using the classic rolling ball algorithm (ball radius = 105 px; chosen empirically to exceed the largest MyHC1+ fiber), and this radius should be adjusted to match cell size in other datasets. The function *calculate_average_intensity* computes the mean pixel intensity from the background‐subtracted image specifically within the coordinates defined by each fiber mask. Fibers with a mean MyHC1+ intensity >20 are classified as type I (assigned value: 1) and those below are type II (assigned value: 0). The threshold value was derived from an intensity analysis of 3722 cells from 12 human biopsies within our data set (Figure 6).

#### 2.7.6 Batch orchestration and outputs

The *process_subject* function automates the workflow across all timepoints and legs for a given subject. The pipeline generates several high‐value outputs:

- *Excel workbooks*: A unified .xlsx file containing sheets for each biopsy, including columns for fiber ID, area, perimeter, nuclei count, satellite cell count, and MyHC1 intensity.
  - Certain identified cells, specifically those labeled as cell number “0”, should be sorted and removed from the data set as the pipeline assigns extraneous nuclei to these cells. Any cells that had 10 or more nuclei assigned to it are manually verified or removed from the spreadsheet.
- *Diagnostic overlays*: Labeled laminin images are generated for rapid visual quality control (QC) to verify the accuracy of the automated counts.

### 2.8 Validation of pipeline

For validation of the method presented here, we included 310 cells from one 23 year old male individual at baseline. This individual was a part of a larger study. For pipeline validation, 310 cells were matched and analyzed both manually and by the pipeline (Mackey et al., 2009). For each cell, borders were traced by hand and myonuclei, Pax7+ satellite cells, and fiber type were scored manually. Area and perimeter of each cell were recorded based on hand drawn tracings. The pipeline produced the same five metrics for the 310 cells. Methods were compared using Bland Altman analyses measuring the absolute difference, paired two‐tailed *t*‐tests (*α* = 0.05), frequency distributions, and correlation analyses.

## 3 RESULTS

### 3.1 Validation of myonuclei per cell

**FIGURE 7:**
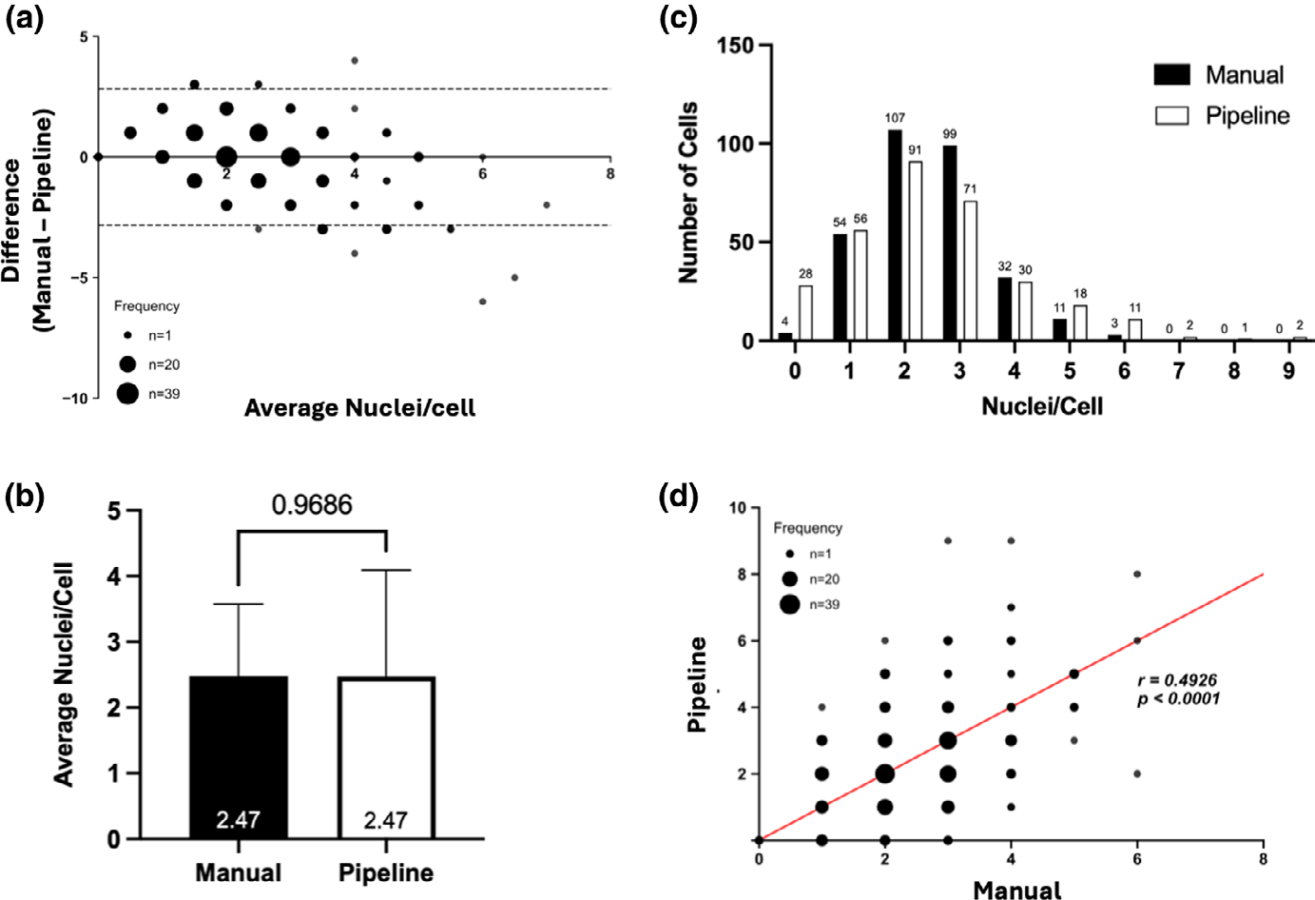
Comparison of nuclei/cell counted manually versus by the pipeline (*N* = 310). (a) Bland Altman 95% limits of agreements plot comparing manual nuclear counting to pipeline nuclear counting. The frequency of data points is represented by point size. (b) Average nuclei/cell as counted by manual analysis and by the pipeline. Paired *t*‐test resulted in a two‐tailed *p*‐value of 0.9686. Data is presented as mean ± standard deviation. (c) Frequency distribution of nuclei/cell for manual (black bars) and pipeline (white bars) analysis. (d) Correlation between nuclei/cell counted by the pipeline and by manual analysis; *r* = 0.4926 and *p* < 0.0001. Red line represents the line of identity.

Bland Altman comparison of manual versus pipeline myonuclei per cell resulted in a bias of −0.003 nuclei with 95% limits of agreement of −2.831 to 2.824 nuclei (Figure 7a). Across 310 cells, the average manual myonuclei/cell was 2.471 and the average pipeline myonuclei/cell was 2.474, with no significant difference by paired *t*‐test (*p* = 0.9686; Figure 7b). Frequency distributions for both methods are shown in Figure 7c. The two methods were moderately correlated (*r* = 0.4926, *p* < 0.0001).

### 3.2 Validation of Pax7+ satellite cells per cell

**FIGURE 8:**
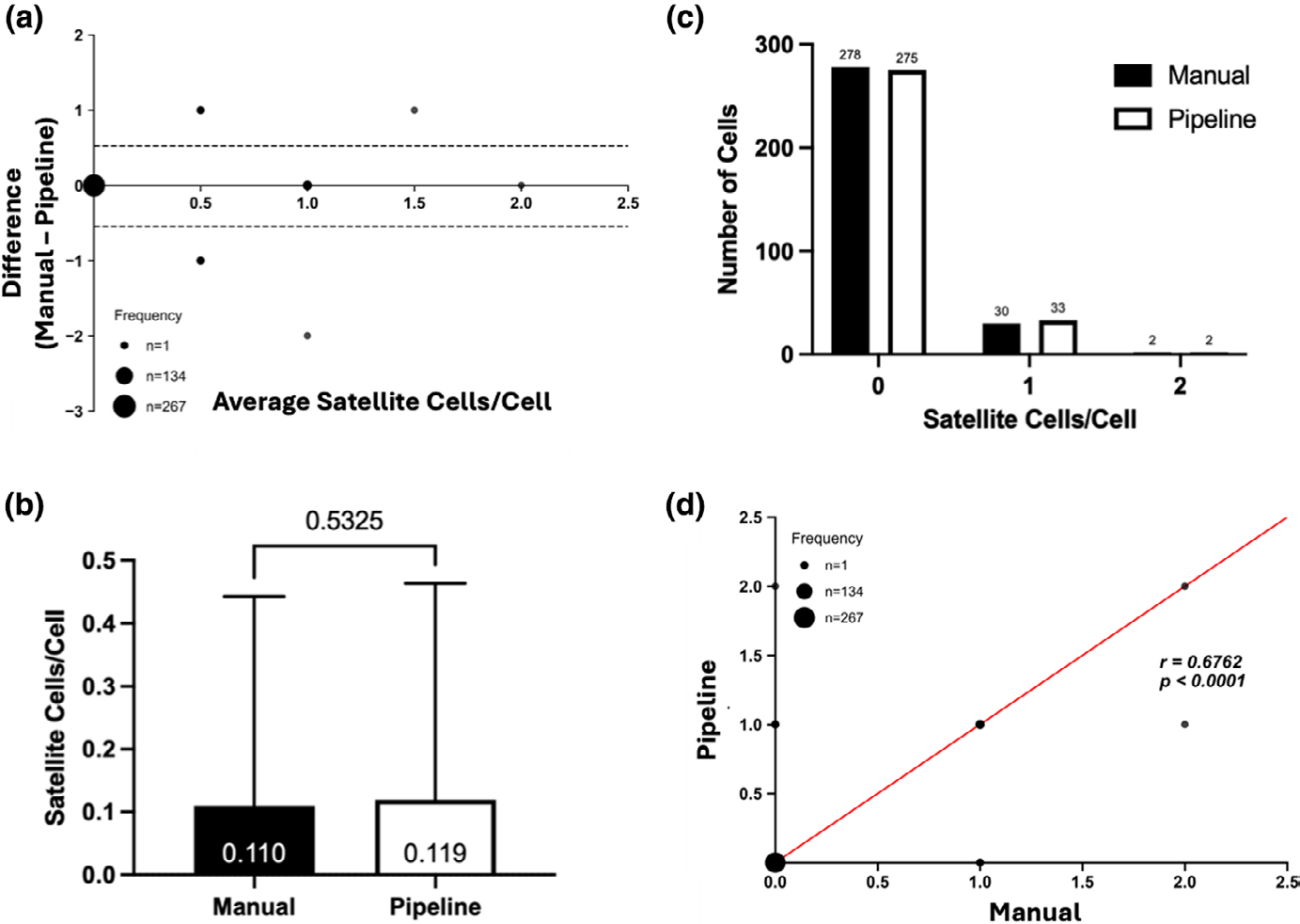
Comparison of satellite cells/cell counted manually versus by the pipeline (*N* = 310). (a) Bland Altman 95% limits of agreements plot comparing manual satellite cell counting to pipeline satellite cell counting. The frequency of data points is represented by point size. (b) Average satellite cells/cell as counted by manual analysis and by the pipeline. Paired *t*‐test resulted in a two‐tailed *p*‐value of 0.5325. Data is presented as mean ± standard deviation. (c) Frequency distribution of satellite cells/cell for manual (black bars) and pipeline (white bars) analysis. (d) Correlation between satellite cells/cell counted by the pipeline and by manual analysis; *r* = 0.6762 and *p* < 0.0001. Red line represents the line of identity.

Bland Altman comparison of manual versus pipeline for Pax7+ satellite cells per cell resulted in a bias of −0.0097 satellite cells with 95% limits of agreement of −0.5441 to 0.5247 satellite cells (Figure 8a). Across the 310 cells, the manual average satellite cells/cell was 0.110 and the pipeline average satellite cells/cell was 0.119, and a paired *t*‐test between the two methods revealed a *p*‐value of 0.5325 (Figure 8b). The frequency distribution shows the range of satellite cells/cell for both the manual and the pipeline methods (Figure 8c). The two methods were moderately correlated (*r* = 0.6762, *p* < 0.0001).

### 3.3 Validation of fiber typing

**FIGURE 9:**
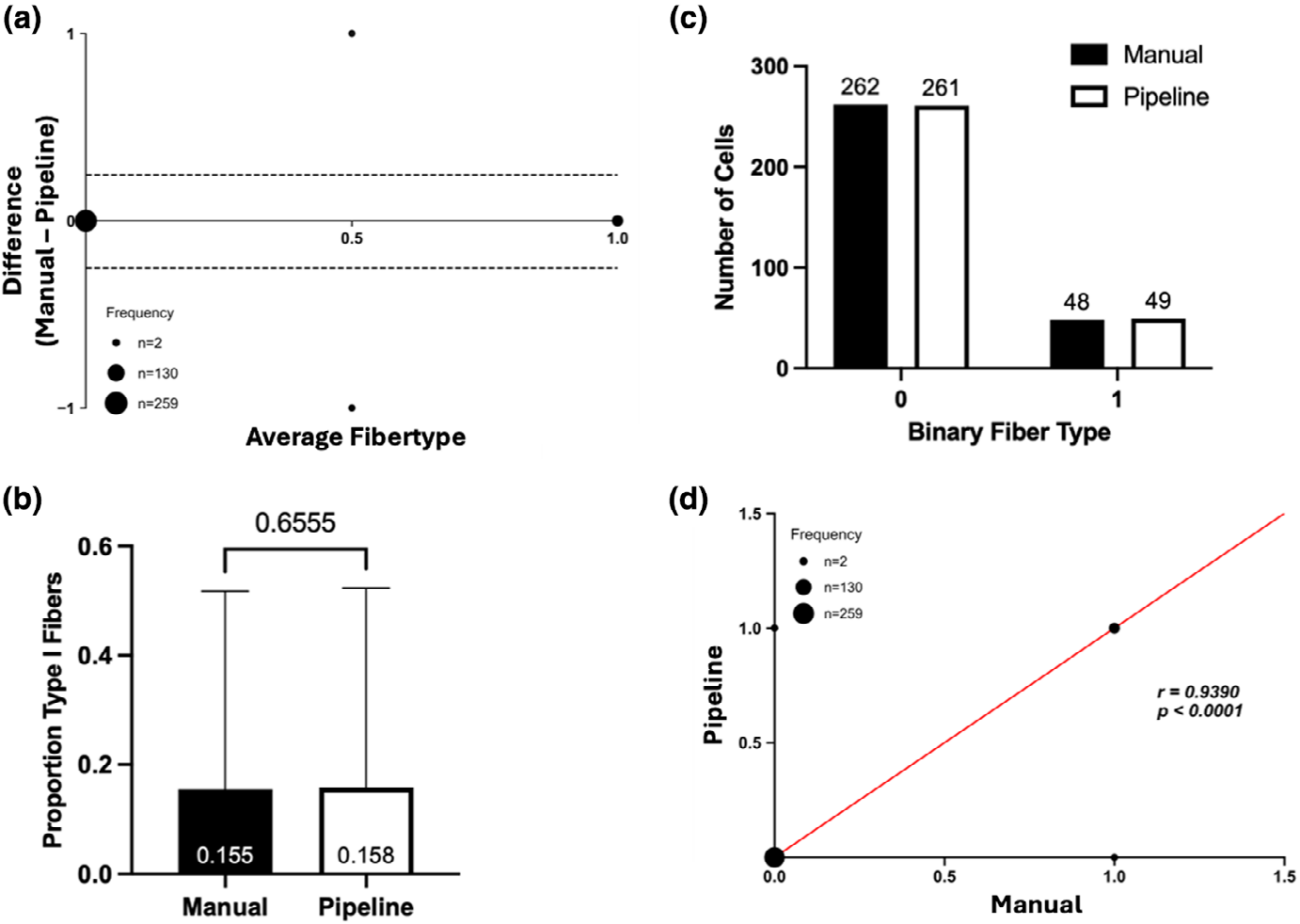
Comparison of fiber type assessed manually versus assessed by the pipeline (*N* = 310). (a) Bland Altman 95% limits of agreements plot comparing manual fiber typing to pipeline fiber typing. The frequency of data points is represented by point size. (b) Proportion of type I fibers as counted by manual analysis and by the pipeline. Paired *t*‐test resulted in a two‐tailed *p*‐value of 0.6555. Data is presented as mean ± standard deviation. (c) Frequency distribution of type I and type II fibers. For manual (black bars) and pipeline. Type I fibers have a binary fiber type of 1 (presence of signal), type II fibers have a binary fiber type of 0 (no signal present). (d) Correlation between pipeline and manual fiber typing; *r* = 0.9390 and *p* < 0.0001. Red line represents the line of identity.

For fiber typing, Bland Altman analysis resulted in a bias of −0.0032 type I cells with 95% limits of agreement of −0.2525 to 0.2460 (Figure 9a). Across 310 cells, the manual proportion of type I fibers was 0.155 manually versus 0.158 for the pipeline, and the paired *t*‐test revealed a *p* value of 0.6555 (Figure 9b). Manual fiber typing identified 48 type I fibers and 262 type II fibers while automated fiber typing identified 49 type I fibers and 261 type II fibers (Figure 9c). The two methods were highly correlated (*r* = 0.9390, *p* < 0.0001).

### 3.4 Validation of cross sectional area measurements

**FIGURE 10:**
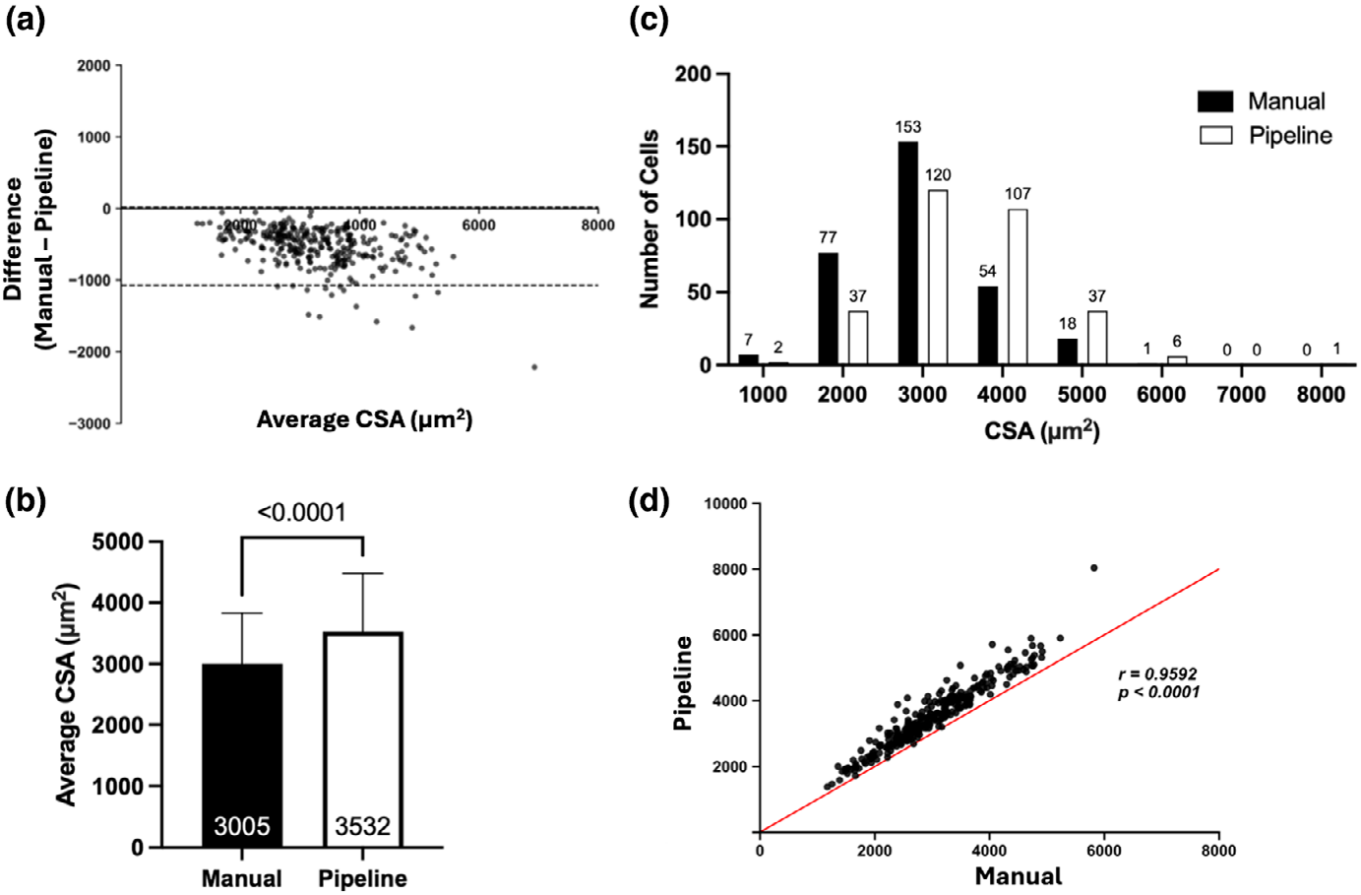
Comparison of cross‐sectional area (CSA) measured manually versus by the pipeline (*N* = 310). (a) Bland Altman 95% limits of agreements plot comparing manual cross‐sectional area to pipeline cross sectional area. (b) Average cross‐sectional area counted by manual analysis and by the pipeline. Paired *t*‐test resulted in a two‐tailed *p*‐value less than 0.0001. Data is presented as mean ± standard deviation. (c) Frequency distribution of cross‐sectional area for manual (black bars) and pipeline (white bars) analysis. (d) Correlation between cross‐sectional area from the pipeline and measured manually; *r* = 0.9592 and *p* < 0.0001. Red line represents the line of identity.

To validate area measurements, the manual outlines measured by Fiji were compared to automated area outputs from CellProfiler. Bland Altman analysis comparing manual and pipeline cross‐sectional area (CSA) measurements showed a bias of −527 μm^2^ with 95% limits of agreement of −1072 to 18.11μm^2^ (Figure 10a). Across the 310 cells, the mean manual CSA was 3005μm^2^ versus 3532μm^2^ for the pipeline, and the paired *t*‐test was significant (*p*‐value <0.0001; Figure 10b). Frequency distributions of CSA for both methods are shown in Figure 10c. The methods were highly correlated (*r* = 0.9592, *p* < 0.0001; Figure 10d).

### 3.5 Validation of perimeter measurements

**FIGURE 11:**
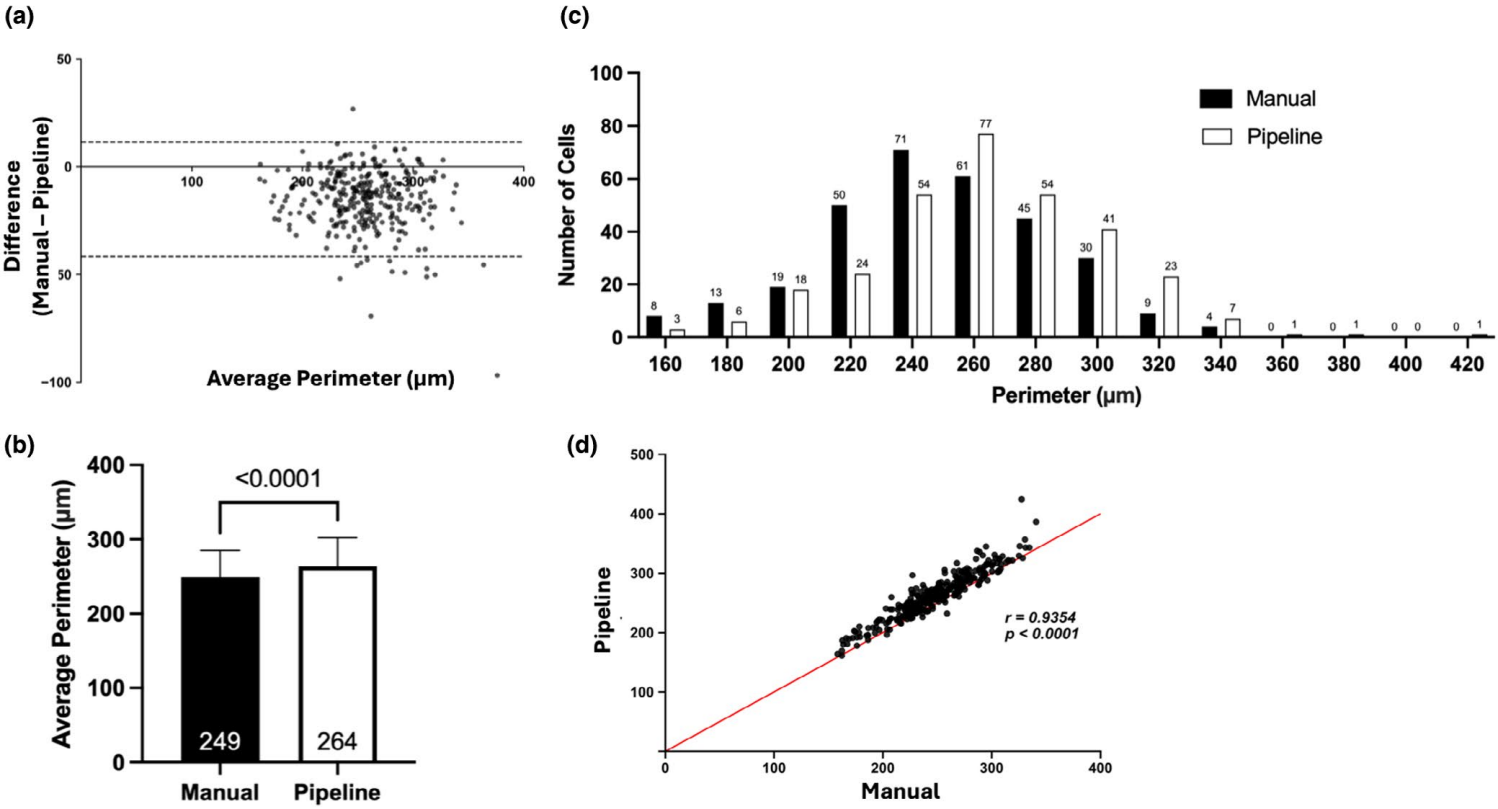
Comparison of perimeter measured manually versus by the pipeline (*N* = 310). (a) Bland Altman 95% limits of agreements plot comparing manual perimeter pipeline perimeter. (b) Average perimeter counted by manual analysis and by the pipeline. Paired *t*‐test resulted in a two‐tailed *p*‐value less than 0.0001. Data is presented as mean ± standard deviation. (c) Frequency distribution of perimeter for manual (black bars) and pipeline (white bars) analysis. (d) Correlation between perimeter from the pipeline and measured manually; *r* = 0.9354 and *p* < 0.0001. Red line represents the line of identity.

For perimeter validation, the perimeters of the manually drawn outlines used for CSA were measured and recorded by Python and compared to automated perimeter outputs from CellProfiler. Bland Altman comparing manual versus pipeline for perimeter resulted in a bias of −15.13 μm with 95% limits of agreement of −41.69 μm to 11.43 μm (Figure 11a). Across the 310 cells, the manual average perimeter was 249 μm versus 264 μm for pipeline, and the paired *t*‐test was significant (*p* < 0.0001; Figure 11b). Frequency distributions are shown in Figure 11c. The methods were highly correlated (*r* = 0.9354, *p* < 0.0001; Figure 11d).

## 4 DISCUSSION

In this study, we developed and validated a semi‐automated pipeline analysis method to quantify Pax7+ satellite cells, myonuclei, cross‐sectional area, and perimeter by fiber type in skeletal muscle cross‐sections. This approach delivers reproducible, unbiased measurements with considerably reduced analysis time and user dependence compared with manual analysis.

While several automated pipelines exist for analyzing muscle tissue ultrastructure (Babcock et al., 2020; Encarnacion‐Rivera et al., 2020; Lau et al., 2018; Mayeuf‐Louchart et al., 2018; Sanz et al., 2019; Wen et al., 2018), none contain our parameters of interest—Pax7+ satellite cells, myonuclei, fiber CSA, perimeter, and fiber type—into a single workflow. Additionally, existing pipelines are dependent on images that are free of extraneous (background) signals or imperfect tissue regions (Wen et al., 2018). Our goal was to build a robust, modular pipeline that quantifies these specific endpoints, while remaining modular enough to be adapted to other tissue types or parameters of interest. For example, within our lab, the pipeline presented here was readily modified to identify macrophages (in place of satellite cells) and atrophied myofibers from an elderly clinical cohort.

Our first aim was accurate quantification of myonuclei per myofiber, which can be difficult due to other cell types present within the muscle cross‐section. By combining Fiji‐based pre‐processing to remove extraneous signal with automated Python analysis, the pipeline yields reproducible nuclear counts that closely match manual scoring. Although the Fiji image cleaning step is the most time‐consuming component, it is essential for reducing false positives and improving confidence in downstream outputs. Overall, the pipeline workflow is substantially faster and far more reproducible than manual analysis.

Our second aim was to achieve accurate quantification of Pax7+ satellite cells per fiber. This was achieved using the highly modular CellProfiler platform. By specifying input size, circularity, and intensity filters tuned to Pax7+ signal, the pipeline reliably discriminated satellite cells from background artifacts.

The third aim was to scale up throughput to quantify hundreds to thousands of muscle cells per cross‐section. Published power estimates reveal that a lower limit of 50 type I and 75 type II fibers is required for comparisons of satellite cells per myofiber (Mackey et al., 2009); historically, we selected a minimum threshold of 150 cells per biopsy. While dependent on biopsy size, our pipeline can process over 1000 cells per sample, substantially increasing sample representation and efficiency.

The pipeline offers several practical advantages beyond its main outputs. CellProfiler's modular design allows for the easy addition of other parameters or the adjustment of current parameters to accommodate different tissue types, antibodies, or imaging conditions (Sanz et al., 2019). The pipeline generates and saves segmentation overlays for each parameter within each image set, and the custom Python script assigns unique identifiers to every cell, features that are crucial for quality control and auditing. Finally, the Python script compiles results into a single Excel file that can be easily sorted for statistical analysis.

### 4.1 Limitations

**FIGURE 12:**
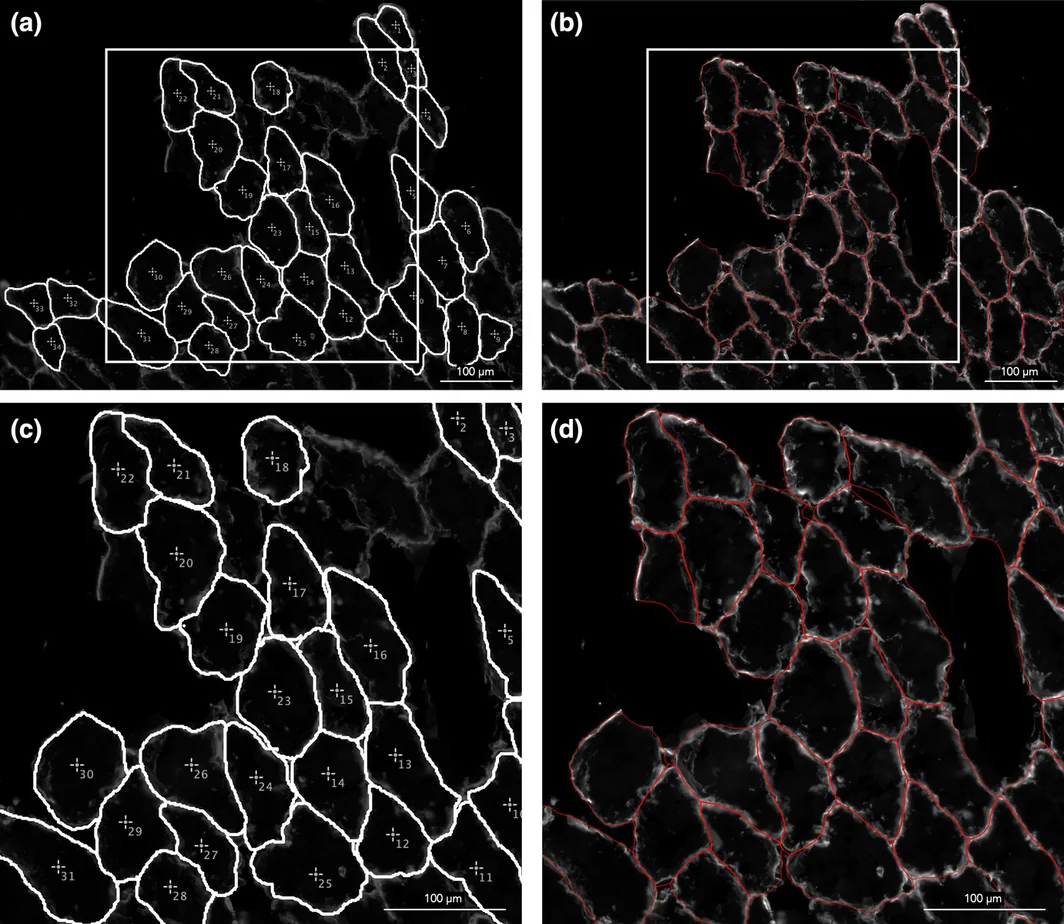
Manual versus CellProfiler cell outlines. (a) Manually drawn outlines shown in white, drawn over the raw laminin stain. Brush width used in Fiji was 10 pixels. Scale bar = 100 μm. (b) Automated outlines shown in red, drawn over the raw laminin stain shown in (a). Line width used by CellProfiler was 1 pixel. Scale bar = 100 μm. (c) Callout from white box in (a). Scale bar = 100 μm. (d) Callout from white box in (b). Scale bar = 100 μm.

While the primary outputs (myonuclei/cell, Pax7+ satellite cells/cell, and fiber type) closely match manual measurement, systematic differences remain for CSA and perimeter. Manual outlines were drawn directly on or slightly within the laminin border, using a 10‐pixel brush in Fiji (Figure 12a,c). In contrast, the pipeline places segmentation on or slightly outside of the laminin border, with a 1‐pixel outline (Figure 12b,d). Because the CSA and perimeter are computed from inside the drawn outline, these small boundary offsets produce measurable differences in calculated area and perimeter. Similar discrepancies between manual and automated fiber outlines have been previously reported (Wen et al., 2018).

Other limitations are the pipeline requires image cleaning and strict file naming before analysis. Although time consuming, removing extraneous artifacts and ensuring clear target visibility substantially improves the accuracy of the final output. Similarly, the strict file naming allows for automated organization of and batch processing of large image sets. As noted above, fibers labeled “fiber 0” include extraneous nuclei not assigned to any fiber—and these cases should be visually verified or excluded prior to analysis.

The *RunCellpose* “nuclei” and “cyto2” models may fail to detect objects in images with very sparse or very dim signal, returning zero nuclei or cells for those fields; this is generally when <10 cells occupy the field of view. The issue can also occur if the DAPI signal is very dim. This issue has been reported in community forums (e.g., GitHub, Image.sc). In our experience sparse images are uncommon, but, if dim, sparse, or noisy images occur frequently, users can try alternative detection modes within *RunCellpose*, or upgrade to the newer version of Cellpose, (e.g., Cellpose3) to improve detection. Finally, this pipeline classifies satellite cells only on the presence or absence of the tyramide‐amplified Pax7 signal. We have manually cross referenced the Pax7 and DAPI channels, and in our hands, the tyramide‐amplified Pax7 signal tags satellite cells that are both Pax7+/DAPI+. However, if users would prefer both Pax7+/DAPI+ classification for satellite cells, code can be added to the python script to cross reference the nuclei masks with the satellite cell masks before counting a satellite cell as a positive hit.

In conclusion, we present a semi‐automated pipeline that (1) quantifies myonuclear number per fiber; (2) quantifies Pax7+ satellite cells per fiber; (3) quantifies CSA and perimeter per fiber; (4) assigns fiber type, and (5) aggregates outputs for quality control and ease of statistical analysis. Designed and optimized for batch processing and organization of large numbers of widefield images, the workflow generates rich datasets describing myonuclear and satellite cell dynamics across entire cross‐sections. This workflow has the potential for broad application in muscle biology and clinical settings alike.

## AUTHOR CONTRIBUTIONS

**Helia G. Megowan:** Conceptualization; data curation; formal analysis; investigation; methodology; validation; visualization. **Madeline Luu:** Conceptualization; data curation; formal analysis; investigation; methodology; validation; visualization. **Adam Shuaib:** Conceptualization; investigation; methodology; visualization. **Kaitlyn B. Augienello:** Data curation; investigation. **Adam C. Fries:** Resources; supervision; validation; visualization. **Jake Searcy:** Supervision. **Hans C. Dreyer:** Conceptualization; funding acquisition; resources; software; supervision.

## FUNDING INFORMATION

This work was supported by the Wu Tsai Human Performance Alliance and the Joe and Clara Tsai Foundation.

## Supporting information


**Data S1.** Supporting Information.

## Data Availability

All data and materials are available in the manuscript and supplemental protocol. Code is available at: https://github.com/DreyerLabUO/23Wu‐CrossSection.
