# Supplementary material for: A semi‐automated pipeline for quantitation of Pax7+, myonuclei, and cross‐sectional area by fiber type
Source: Physiol Rep. 2026 Sep 21;14(18):e71097. doi: 10.14814/phy2.71097 (PMC13591552; doi:10.14814/phy2.71097)
Supplement: Supplementary file 1 [file PHY2-14-e71097-s001.pdf]

Supplemental Documentation

Table of Contents

I. **Image Acquisition and Pre-Processing**
   a. **Image processing in *FIJI/ImageJ***
II. **Cross-sectional Myofiber Object Segmentation with *CellProfiler***
   a. **Verifying pipeline with *CellProfiler* GUI**
   b. **Running pipeline with CellProfiler GUI**
   c. **Running pipeline with CellProfiler headless**
III. **Analysis with Python**
   a. **Running the script**
   b. **Output Files and Their Interpretation**
IV. **Our Computer Specifications**
V. **CellProfiler Pipeline Parameters**

I. Image Acquisition & Pre-Processing

A) *Image processing with FIJI/ImageJ*

Setup:

1. Install "*FIJI with Batteries"* from web browser.

Procedure:

1. **Image Acquisition and Initial Standardization**

The dataset for each sample must consist of four channels: Laminin, DAPI, MyHC1, and Pax7.

1. Selection: Evaluate all available images. If duplicate DAPI channels exist, select the image with the highest signal-to-noise ratio. If neither is superior, both may be retained for later merging.
2. File Formatting: All images must be saved in .tif format.
3. Preliminary Renaming: Before processing in FIJI, rename files to ensure compatibility with the *CellProfiler* pipeline. Alternative nomenclatures can be used, but will require modification of metadata parsing pattern in *CellProfiler* pipeline.
   - Remove technical strings (e.g., replace _ch00 with a blank space).
   - Abbreviate "Baseline" to BL and "Laminin" to Lam.
   - Standard Nomenclature: SubjectID_Timepoint_Side_Set#_Channel (e.g., Subject8_BL_L_Set1_Lam).

2. Preparation of the MyHC1 Channel

The MyHC1 image serves as a reference and does not require manual cleaning.

- Rename the MyHC1 file according to the protocol in Step 1.
- Transfer the file directly to the designated "Cleaned Images" output directory.

3. Contrast Adjustment and Channel Merging (FIJI/ImageJ)

Import the Laminin, Pax7, and DAPI images into FIJI.

1. Optimization: If necessary, adjust visualization parameters via Image > Adjust > Brightness/Contrast. Modify the minimum and maximum sliders to enhance feature definition. These adjustments are for visualization during cleaning and do not require separate saving.
2. Bit-Depth Verification: Ensure all channels are consistent (e.g., 8-bit). If a "bit-depth" error occurs, convert the image via Image > Type > 8-bit.
3. Channel Merging: To facilitate simultaneous cleaning across channels, merge the images via Image > Color > Merge Channels.

- Assign arbitrary colors to each channel based on visual clarity.
- Crucial: Enable the "Keep source images" option to preserve the original single-channel data.

4. Manual Artifact Removal ("Cleaning")

Artifacts, such as tissue folds or non-specific staining, must be removed to prevent errors in downstream segmentation.

1. Selection: Use the Freehand Selection Tool to circumscribe the artifact.
2. Deletion: Navigate to Edit > Clear to remove the signal within the selection.
   - *Warning:* Do not use "Clear Outside," as this action is destructive to the rest of the image data.
3. Iteration: Continue this process until all significant artifacts are removed from the merged view.

5. Export and Directory Structure

1. Channel Splitting: Once cleaning is complete, revert the merged image to individual channels via Image > Color > Split Channels.
2. Saving: Export each cleaned channel via File > Save As > Tiff. Adhere strictly to the naming convention established in Step 1.
3. Data Organization: Organize cleaned images into biopsy-specific folders using the following format: sub[ID]_[Timepoint]_[Side]_clean (e.g., sub8_BL_L_clean). This structure is required for the CellProfiler input module.

Rationale: Image processing is a necessary step towards a smooth analysis pipeline. A clean image has is free of "abnormal regions" to produce reliable and high-quality data. We remove sections where Laminin is blurry or crossed over itself, where DAPI is not clear, and overall, any section that we would not count as cells in traditional manual analysis.

Adaptations:

- Modify contrast adjustment and/or artifact removal in accordance with immunohistochemistry methods used.
- Adjust file naming conventions in compatibility with your project. Adjustments in file naming conventions at this step will require downstream adaptations to *CellProfile*r and Python script pipeline.

II. Cross-sectional Myofiber Object Segmentation with *CellProfiler*

1. *Verifying pipeline with* CellProfiler *GUI*

Before full-scale deployment, the modular pipeline must be validated within the *CellProfiler* Graphic User Interface (GUI) to ensure that segmentation parameters, specifically deep-learning thresholds and intensity-based filters, are optimized for the specific optical characteristics of the dataset.

Setup:

1. Install **CellProfiler v4.2.8** and **Docker Desktop** from web browser. Docker is required to host the Cellpose container, ensuring a stable environment for the `cyto2` and `nuclei` models.
2. **Plugin Configuration**: Download the `runcellpose.py` module from the CellProfiler-plugins repository (https://github.com/CellProfiler/CellProfiler-plugins) and move it to the local plugins directory. In *CellProfiler*, navigate to `Preferences` and set the **Plugins Directory** to this path.
3. **Data Input**: Load the "Cleaned Images" directory (generated in Section I) into the **Images** module.
4. **Pipeline Initialization**: Import the `.cppipe` file from the Dreyer Lab GitHub repository to load the pre-configured modular architecture.

Procedure:

1. **Metadata and Channel Assignment**: In the **Metadata** module, update the Regular Expression (RegEx) to parse subject and biopsy identifiers. Use the **NamesAndTypes** module to assign tags based on naming conventions (e.g., Lam to "laminin", DAPI to "nuclei").
2. **Fiber Segmentation Review**: Navigate to the **RunCellpose** module assigned to the Laminin channel. Verify that the 'cyto2' model is selected and that the resulting outlines align precisely with the sarcolemma.
3. **Iterative Nuclear Refinement**:
   - Step through the **GaussianFilter** (sigma = 3) to confirm the smoothing of the DAPI signal.
   - Examine the **ImageMath** module to verify that the subtraction of the 17th percentile of pixel intensities effectively suppresses low-level background fluorescence and non-specific staining.
   - Inspect the final **RunCellpose** ('nuclei' model) output using a flow_threshold of 0.4 and cellprob_threshold of 0.0 to ensure precise myonuclear boundary adherence.
4. **Satellite Cell Identification**: In the **IdentifyPrimaryObjects** module, confirm that the **Otsu thresholding** and pixel diameter range (16–60 pixels) accurately isolate Pax7+ signals from the sarcoplasmic background.
5. **Diagnostic Visualization**: Execute **Test Mode** and use the "Window Display" to view red-line overlays on raw signals, ensuring no significant over- or under-segmentation occurs before proceeding to headless batch processing.

Rationale: Direct verification in the GUI is a critical quality control step that allows researchers to visually calibrate modular variables against the biological variability of the tissue. By manually inspecting the segmentation of the first few image sets, users can ensure that the deep-learning models and background subtraction logic are performing with high fidelity, minimizing the risk of false-positive nuclear assignments or inaccurate cross-sectional area (CSA) measurements during high-throughput execution.

Adaptations:

- **Signal Intensity Variations**: If the DAPI signal is consistently dim across a cohort, the subtraction percentile in the **ImageMath** module should be reduced (e.g., to the 10th percentile) to prevent the loss of true myonuclear signals.
- **Object Size Constraints**: Adjust the pixel diameter range in the **IdentifyPrimaryObjects** module for satellite cells if the imaging magnification or species-specific cell sizes differ from the human vastus lateralis parameters provided here.
- **Alternative Detection Modes**: For images with extremely high noise or sparse signals, users may test the newest Cellpose models or alternative detection modes within the **RunCellpose** module to improve boundary detection.

2. *Running pipeline with* CellProfiler *GUI*

Following successful parameter verification, the high-throughput batch processing workflow is executed within the CellProfiler GUI to automate cellular segmentation and feature extraction across large datasets.

Setup

1. Launch Docker Desktop and *CellProfiler*. Open CellProfiler pipeline (.cpproj).
2. **Input Directory Structure**: All "Cleaned Images" must be organized into biopsy-specific folders (e.g., `sub[ID]_[Timepoint]_[Side]_clean`) to facilitate the **Metadata** module's parsing logic.
3. **Default Output Folder**: Set the global output directory where the pipeline will programmatically generate the hierarchical folder structure for masks and morphological data.

Procedure

1. **Batch Initiation**: Click the **Analyze Images** button to start sequential processing. The **Groups** module will systematically organize image sets by Subject, Day, Side, and Set number based on previously extracted metadata.
2. **Sequential Segmentation Streams**:
    - **Fiber Masks**: The system invokes the Cellpose 'cyto2' model to generate binarized sarcolemmal masks from the Laminin channel.

- **Nuclear Masks**: The pipeline executes the background-subtraction logic—subtracting the 17th percentile of pixel intensities—followed by the Cellpose 'nuclei' model to produce refined myonuclear masks.
- **Satellite Cell Masks**: **IdentifyPrimaryObjects** isolates Pax7+ signals using the established Otsu thresholding and size constraints (16–60 pixels).
- **MyHC1 images**: MyHC1 images are organized into desired directory location alongside output masks for downstream analysis.

3. **Data Serialization**: CellProfiler saves grayscale object masks (label matrices) and diagnostic overlays for every channel in each image set. These outputs are organized into subdirectories (e.g., laminin_masks, nuclei_masks, satcell_masks) for downstream Python analysis.

Rationale: Running the pipeline through the GUI provides a semi-automated balance between high-throughput speed and rigorous quality control. This environment allows the researcher to monitor segmentation progress in real-time and ensures that the standardized hierarchical directory structure is maintained, which is a prerequisite for the custom Python processing script. While the pipeline is highly automated, manual verification of the final diagnostic overlays is maintained to correct for any missed or artificial identifications, particularly in tissue regions with variable staining quality.

Adaptations

- **High-Volume Processing**: For datasets exceeding several hundred image sets, users should transition to the **Headless/CLI** mode (Section II-C) to reduce memory overhead associated with GUI rendering.
- **Threshold Refinement**: If the **IdentifyPrimaryObjects** module fails to capture sparse Pax7+ signals during a run, the intensity threshold can be manually overridden in the module settings to accommodate specific batch variations.
- **Alternative Identification**: This modular workflow can be easily adapted to identify other markers (e.g., macrophages) by swapping the Pax7+ **IdentifyPrimaryObjects** module with a target-specific segmentation block.

3. *Running pipeline with* CellProfiler headless

For high-throughput processing of large-scale cohorts or deployment on high-performance computing (HPC) clusters, the pipeline is executed in "headless" mode via the Command Line Interface (CLI). This method bypasses the Graphical User Interface (GUI) to minimize memory overhead and ensure strictly reproducible batch orchestration.

Setup

1. **Environment Configuration:** Headless execution is supported either through a **Docker container** (v28.2.2) for desktop stability or within a localized **Conda environment** for maximum performance on HPC clusters.
2. **Dependency Installation:** Create and activate a dedicated environment (Python 3.12) (Anaconda Prompt recommended) and install the core analytical suite:

```
conda create --name csapipeline python=3.12

conda activate csapipeline

conda install cellprofiler=4.2.8

python -m pip install numpy pandas scikit-image scikit-learn openpyxl
Pillow
```

3. **Plugin Pathing**: Ensure the `runcellpose.py` plugin is located in a dedicated directory accessible to the CLI.

Procedure:

1. **Command Invocation**: Execute the analysis using the `--run` (`-r`) and `--headless` (`-c`) flags. A standard execution string for the lab workstation follows:

```
& "C:\Program Files\CellProfiler\CellProfiler.exe" -c -r \
--log-level DEBUG \
-p [PIPELINE.cppipe] \
-i [INPUT_FOLDER] \
-o [OUTPUT_FOLDER] \
--plugins-directory=[ACTIVE_PLUG-INS_FOLDER]
```

2. **Metadata Parsing**: The input directory path (`-i`) must point to the root directory containing biopsy-specific subfolders. The CLI automatically applies the Regular Expression (RegEx) patterns defined in the .cpproj pipeline to organize masks and metadata.

Rationale: Headless execution is a mechanical necessity for high-throughput bioinformatics, as it eliminates the memory cost associated with real-time image rendering. This modality provides a superior platform for processing over 1,000 cells per biopsy, ensuring that identical parameters, such as the GaussianFilter (sigma = 3) and the 17th percentile background subtraction, are

applied with absolute consistency across the entire dataset. Furthermore, native Python execution on high-performance clusters yields optimal processing speeds for multi-terabyte datasets compared to traditional desktop environments.

Adaptations

- **Scaling and Magnification**: While the standard pipeline is optimized for 20x magnification, researchers utilizing 10x or 40x objectives must adjust the "Estimated XY Diameter" within the RunCellpose modules to prevent over- or under-segmentation.
- **Fiber Typing Sensitivity**: The MyHC1 intensity threshold is set at a default of 25 in the downstream Python script, but this can be adjusted within the supplemental protocol if specific staining batches exhibit higher background fluorescence.
- **Marker Flexibility**: The modular design allows for the replacement of the Pax7+ identification module with other markers, such as macrophage-specific antibodies, by adjusting the pixel diameter range and thresholding strategy in the *IdentifyPrimaryObjects* module.

III. Analysis with *Python*

A) Run the Python Analysis

The final stage of the workflow transitions from raw segmentation masks to refined, biologically relevant datasets through a custom Python-based processing pipeline. This pipeline automates the integration of multi-channel spatial data, ensuring objective fiber-type assignment and high-throughput quantification.

Setup

1. **Environment Initialization:** Create and activate a dedicated environment (Python 3.12) (Anaconda Prompt recommended) and install the core analytical suite:

   ```
   conda create --name csapipeline python=3.12
   conda activate csapipeline
   python -m pip install numpy pandas opencv-python XlsxWriter
   ```

2. **Script Preparation**: Save the execution script, `23_WU_CSA_ANALYSIS.py`, in your primary working directory.
3. **Input Organization**: Ensure that the outputs from CellProfiler are organized into a strict hierarchical directory structure:

   ```
   Subject/
             Timepoint/
                 L or R/
                    laminin_masks/
                    nuclei_masks/
                    satcell_masks/
                    MyHC1_images/
                    …
   ```

Procedure:

1. Run the pipeline. From the activated Conda environment terminal, run the script with desired arguments:

   ```
   python 23_WU_CSA_ANALYSIS.py <input_dir> <output_dir> <subject_name>
   ```

Rationale: The custom Python pipeline is a mechanical necessity to bridge the gap between computer vision outputs and biological relevance. By utilizing "greatest overlap" logic, the pipeline maintains high-fidelity assignments even in irregular or compressed fiber morphologies. Furthermore, automating fiber-type assignment via the `calculate_average_intensity` function removes the subjectivity inherent in manual fluorescence intensity estimation, ensuring that classification is strictly based on quantified signal distributions.

Adaptation:

- **Scaling Factors**: Users must adjust the scaling constant within the `calculate_area_and_perimeter` function if imaging magnification differs from the 20x standard used in this study.
- **Rolling Ball Radius**: While the rolling ball radius for background subtraction is set at 100 pixels, users should adjust this value to exceed the largest cell, in cross-sectional area, present in the dataset.
- **Threshold Calibration**: While the Type I intensity threshold is set at 20, users should perform a distribution analysis (as seen in Figure 6 of the manuscript) to determine the optimal "elbow" for different staining batches.
- **Naming Conventions**: The dynamic directory parser relies on the naming convention established during pre-processing; any modifications to file nomenclature in FIJI will require corresponding updates to the RegEx patterns within the Python script (as with the CellProfiler pipeline).

B) Output Files and Their Interpretation

The pipeline yields two primary data structures for research application and quality control (QC):

- **Subject Workbooks**: A unified Excel (.xlsx) file is generated for each subject, containing individual sheets per biopsy. Each sheet provides a morphological profile for every segmented fiber, including unique Fiber ID, Cross-Sectional Area (CSA), Perimeter, Myonuclear count, Satellite Cell count, and binary Fiber Type classification.

- **Diagnostic Overlays**: Labeled Laminin masks are saved as .tiff files to allow for rapid visual QC. These images feature unique numerical identifiers for every fiber, enabling researchers to cross-reference quantitative data with original histology.

- **Data Interpretation and Filtration**: During final analysis, users must sort and remove data labeled as "Fiber 0," as this category represents extraneous assignments. Furthermore, any fiber assigned more than 10 nuclei must be manually verified using the diagnostic overlays to ensure segmentation accuracy.

IV. Our Computer Specifications

This pipeline was trained and developed on both a standard, in-lab desktop workstation and a high-performance computer operated in the Genomics and Cell Characterization Core Facility (G3CF) at the University of Oregon.

***Lab desktop*:** Dell Precision 3630 Tower workstation running Microsoft Windows 11 Enterprise (Build 26100). The system is equipped with an Intel® Core™ i7-9700 CPU @ 3.00 GHz with 8 cores and 8 threads and operates in UEFI mode with Secure Boot enabled. The machine has a 64-bit architecture and BIOS version 2.26.0 (dated December 8, 2023).

***G3CF computer*:** 32-core AMD Ryzen Threadripper 3970X processor running at 3.9 GHz and 256 GB of DDR4 RAM. The system features a custom water-cooled configuration optimized for high-throughput image processing and is powered by an NVIDIA GeForce RTX 5090 graphics card to support GPU-accelerated rendering and analysis tasks. The machine runs Microsoft Windows 10 Enterprise (version 10.0.19045, build 19045) in UEFI BIOS mode. The software environment includes Imaris 9.9 with the Cell and Filaments Modules Imaris 9.9 Converter, MATLAB 2018b, and Zeiss Zen Black.

V. *CellProfiler* Pipeline Module Parameters

Pipeline: 23_WU_CSA_PIPELINE.cpproj | CellProfiler 4.2.8

Step	Module	Parameter	Value
**#01**	**RunCellpose**<br>*Cellpose for laminin fibers*	Rescale images before running Cellpose	Yes ●
		Run CellPose in docker or local python environment	Docker
		Select Cellpose docker image	cellprofiler/runcellpose_with_pretrained:2.3.2
		Detection mode	cyto2
		Supply nuclei image as well?	No ●
		Select the input image	laminin
		Expected object diameter	180
		Cell probability threshold	0.0
		Minimum size	40
		Flow threshold	0.4
		Name the output object	laminin_identified
		Invert images	No ●
		Save probability image?	No ●
		Use 3D	No ●
		Stitch Threshold	0.0
**#02**	**ConvertObjectsToImage**	Select the input objects	laminin_identified
		Name the output image	laminin_identified_mask
		Select the color format	Grayscale
**#03**	**SaveImages**	Select the type of image to save	Image

Step	Module	Parameter	Value
		Select the image to save	laminin_identified_mask
		Select method for constructing file names	From image filename
		Select image name for file prefix	laminin (from NamesAndTypes)
		Append a suffix to the image file name?	Yes ●
		Text to append to the image name	_laminin_masks
		Saved file format	tiff
		Image bit depth	8-bit integer
		Save with lossless compression?	Yes ●
		Output file location	Default Output Folder sub-folder
		Sub-folder	./Subject/Day/Side/laminin_masks
		Overwrite existing files without warning?	No ●
		When to save	Every cycle
		Record the file and path information to the saved image?	No ●
		Create subfolders in the output folder?	No ●
**#04**	**OverlayOutlines**<br>*Overlays Fibers identified with laminin*	Display outlines on a blank image?	No ●
		Select image on which to display outlines	laminin (from NamesAndTypes)
		Name the output image	laminin_fiber_image
		Outline display mode	Color
		How to outline	Inner
		Select objects to display	laminin_identified (from RunCellpose)
		Select outline color	Red
**#05**	**SaveImages**	Select the type of image to save	Image

Step	Module	Parameter	Value
	Saves laminin and identified fiber overlay		
		Select the image to save	laminin_fiber_image (from OverlayOutlines)
		Select method for constructing file names	From image filename
		Select image name for file prefix	laminin (from NamesAndTypes)
		Append a suffix to the image file name?	Yes ●
		Text to append to the image name	_laminin_fibers
		Saved file format	tiff
		Image bit depth	8-bit integer
		Save with lossless compression?	Yes ●
		Output file location	Default Output Folder sub-folder
		Sub-folder	./Subject/Day/Side/laminin_overlay
		Overwrite existing files without warning?	No ●
		When to save	Every cycle
		Record the file and path information to the saved image?	No ●
		Create subfolders in the output folder?	No ●
**#06**	**GaussianFilter**<br>*GaussianFilter on nuclei image*	Select the input image	nuclei (from NamesAndTypes)
		Name the output image	gaussian_nuclei
		Sigma	3
**#07**	**RunCellpose**<br>*Identified nuclei in the DAPI image*	Rescale images before running Cellpose	Yes ●
		Run CellPose in docker or local python environment	Docker

Step	Module	Parameter	Value
		Select Cellpose docker image	cellprofiler/runcellpose_with_pretrained:2.3.2
		Detection mode	nuclei
		Select the input image	gaussian_nuclei (from GaussianFilter)
		Expected object diameter	15
		Cell probability threshold	0.0
		Minimum size	20
		Flow threshold	0.4
		Name the output object	cellpose_nuclei
		Invert images	No ●
		Save probability image?	No ●
		Use 3D	No ●
		Stitch Threshold	0.0
**#08**	**MeasureImageIntensity**	Select images to measure	gaussian_nuclei
		Measure the intensity only from areas enclosed by objects?	Yes ●
		Select input object sets	cellpose_nuclei
		Calculate custom percentiles	Yes ●
		Specify percentiles to measure	15, 16, 17
**#09**	**ImageMath**<br>*Subtract 12th percentile of intensity from gaussian image*	Operation	Subtract
		Name the output image	_subtracted_gaussian_nuclei
		Image or measurement? (1st operand)	Image
		Select the first image	gaussian_nuclei (from GaussianFilter)

Step	Module	Parameter	Value
		Multiply the first image by	1.0
		Image or measurement? (2nd operand)	Measurement
		Category	Intensity
		Measurement: Measurement	Percentile_17
		Measurement: Image	gaussian_nuclei_cellpose_nuclei
		Multiply the second image by	1.0
		Raise the power of the result by	1.0
		Multiply the result by	1.0
		Add to result	0.0
		Set values less than 0 equal to 0?	Yes ●
		Set values greater than 1 equal to 1?	Yes ●
		Replace invalid values with 0?	Yes ●
**#10**	**RunCellpose**<br>*Rerun Cellpose on subtracted Gaussian image*	Rescale images before running Cellpose	Yes ●
		Run CellPose in docker or local python environment	Docker
		Select Cellpose docker image	cellprofiler/runcellpose_with_pretrained:2.3.2
		Detection mode	nuclei
		Select the input image	_subtracted_gaussian_nuclei (from ImageMath)
		Expected object diameter	25
		Cell probability threshold	0.0
		Minimum size	30
		Flow threshold	0.4

Step	Module	Parameter	Value
		Name the output object	cellpose_subtracted_nuclei
		Invert images	No ●
		Save probability image?	No ●
		Use 3D	No ●
		Stitch Threshold	0.0
**#11**	**ConvertObjectsToImage**	Select the input objects	cellpose_subtracted_nuclei
		Name the output image	filtered_nuclei
		Choose the color format	Grayscale
**#12**	**SaveImages**<br>*Save filtered_nuclei image*	Select the type of image to save	Image
		Select the image to save	filtered_nuclei (from ConvertObjectsToImage)
		Select method for constructing file names	From image filename
		Select image name for file prefix	nuclei (from NamesAndTypes)
		Append a suffix to the image file name?	Yes ●
		Text to append to the image name	_filtered_nuclei
		Saved file format	tiff
		Image bit depth	8-bit integer
		Save with lossless compression?	Yes ●
		Output file location	Default Output Folder sub-folder
		Sub-folder	./Subject/Day/Side/filtered_nuclei
		Overwrite existing files without warning?	Yes ●
		When to save	Every cycle

Step	Module	Parameter	Value
		Record the file and path information to the saved image?	No ●
		Create subfolders in the output folder?	No ●
**#13**	**ConvertObjectsToImage** *Convert nuclei objects to image (nuclei_masks)*	Select the input objects	cellpose_subtracted_nuclei
		Name the output image	nuclei_masks
		Select the color format	Grayscale
**#14**	**SaveImages** *Save nuclei_masks image*	Select the type of image to save	Image
		Select the image to save	nuclei_masks (from ConvertObjectsToImage)
		Select method for constructing file names	From image filename
		Select image name for file prefix	nuclei (from NamesAndTypes)
		Append a suffix to the image file name?	Yes ●
		Text to append to the image name	_nuclei_masks
		Saved file format	tiff
		Image bit depth	8-bit integer
		Save with lossless compression?	Yes ●
		Output file location	Default Output Folder sub-folder
		Sub-folder	./Subject/Day/Side/nuclei_masks
		Overwrite existing files without warning?	No ●
		When to save	Every cycle
		Record the file and path information to the saved image?	No ●

Step	Module	Parameter	Value
		Create subfolders in the output folder?	No ●
**#15**	**OverlayOutlines**<br><br>*cleaned/raw DAPI and nuclei identified overlay*	Display outlines on a blank image?	No ●
		Select image on which to display outlines	nuclei
		Name the output image	nuclei_over_dapi
		Outline display mode	Color
		How to outline	Inner
		Select objects to display	cellpose_subtracted_nuclei
		Select outline color	Red
**#16**	**SaveImages**<br><br>*Cleaned/raw DAPI and nuclei identified overlay*	Select the type of image to save	Image
		Select the image to save	nuclei_over_dapi (from OverlayOutlines)
		Select method for constructing file names	From image filename
		Select image name for file prefix	nuclei (from NamesAndTypes)
		Append a suffix to the image file name?	Yes ●
		Text to append to the image name	nuclei_over_dapi
		Saved file format	tiff
		Image bit depth	8-bit integer
		Save with lossless compression?	Yes ●
		Output file location	Default Output Folder sub-folder
		Sub-folder	./Subject/Day/Side/nuclei_overlay
		Overwrite existing files without warning?	No ●
		When to save	Every cycle

Step	Module	Parameter	Value
		Record the file and path information to the saved image?	No ●
		Create subfolders in the output folder?	No ●
#17	**GaussianFilter**<br>*GaussianFilter on satellite image*	Select the input image	satellite (from NamesAndTypes)
		Name the output image	gaussian_satellite
		Sigma	2
#18	**IdentifyPrimaryObjects**	Use advanced settings?	Yes ●
		Select the input image	gaussian_satellite (from GaussianFilter)
		Name the primary objects to be identified	satcells
		Typical diameter of objects, in pixel units (Min, Max)	16, 60
		Discard objects outside the diameter range?	No ●
		Discard objects touching the border of the image?	Yes ●
		Threshold strategy	Global
		Thresholding method	Otsu
		Two-class or three-class thresholding?	Three classes
		Assign pixels in the middle intensity class to the foreground or the background?	Background
		Threshold smoothing scale	0
		Threshold correction factor	1.0
		Lower and upper bounds on threshold	0.24 1.0
		Log transform before thresholding?	No ●
		Method to distinguish clumped objects	Intensity

Step	Module	Parameter	Value
		Method to draw dividing lines between clumped objects	Intensity
		Automatically calculate size of smoothing filter for declumping?	Yes ●
		Automatically calculate minimum allowed distance between local maxima?	Yes ●
		Speed up by using lower-resolution image to find local maxima?	No ●
		Display accepted local maxima?	No ●
		Fill holes in identified objects?	After both thresholding and declumping
**#19**	**OverlayOutlines**<br>*Satellite cell overlay on satellite image*	Display outlines on a blank image?	No ●
		Select image on which to display outlines	satellite (from NamesAndTypes)
		Name the output image	satcell_overlay
		Outline display mode	Color
		How to outline	Inner
		Select objects to display	satcells (from IdentifyPrimaryObjects)
		Select outline color	Red
**#20**	**SaveImages**<br>*Save satcell_overlay*	Select the type of image to save	Image
		Select the image to save	satcell_overlay (from OverlayOutlines)
		Select method for constructing file names	From image filename
		Select image name for file prefix	satellite (from NamesAndTypes)
		Append a suffix to the image file name?	No ●
		Text to append to the image name	(empty)
		Saved file format	tiff

Step	Module	Parameter	Value
		Image bit depth	8-bit integer
		Save with lossless compression?	Yes ●
		Output file location	Default Output Folder sub-folder
		Sub-folder	./Subject/Day/Side/satcell_outlines
		Overwrite existing files without warning?	Yes ●
		When to save	Every cycle
		Record the file and path information to the saved image?	No ●
		Create subfolders in the output folder?	No ●
**#21**	**ConvertObjectsToImage**<br>*Convert satcells to satcell_masks (Grayscale)*	Select the input objects	satcells from IdentifyPrimaryObjects)
		Name the output image	satcell_masks
		Select the color format	Grayscale
**#22**	**SaveImages**<br>*Save satcell_masks image*	Select the type of image to save	Image
		Select the image to save	satcell_masks (from ConvertObjectsToImage)
		Select method for constructing file names	From image filename
		Select image name for file prefix	satellite (from NamesAndTypes)
		Append a suffix to the image file name?	Yes ●
		Text to append to the image name	_satcell_masks
		Saved file format	tiff
		Image bit depth	8-bit integer
		Save with lossless compression?	Yes ●

Step	Module	Parameter	Value
		Output file location	Default Output Folder sub-folder
		Sub-folder	./Subject/Day/Side/satcell_masks
		Overwrite existing files without warning?	Yes ●
		When to save	Every cycle
		Record the file and path information to the saved image?	No ●
		Create subfolders in the output folder?	No ●
**#23**	**SaveImages**<br>*Save MyHC1 images*	Select the type of image to save	Image
		Select the image to save	MyHC1 (from NamesAndTypes)
		Select method for constructing file names	From image filename
		Select image name for file prefix	MyHC1 (from NamesAndTypes)
		Append a suffix to the image file name?	Yes ●
		Text to append to the image name	(empty — no suffix text entered)
		Saved file format	tiff
		Image bit depth	8-bit integer
		Save with lossless compression?	Yes ●
		Output file location	Default Output Folder sub-folder
		Sub-folder	./Subject/Day/Side/MyHC1_images
		Overwrite existing files without warning?	Yes ●
		When to save	Every cycle
		Record the file and path information to the saved image?	No ●
		Create subfolders in the output folder?	No ●
